# Mixed Ligand Complexes of *N*-Methyl-*N*-phenyl Dithiocarbamate: Synthesis, Characterisation, Antifungal Activity, and Solvent Extraction Studies of the Ligand

**DOI:** 10.1155/2015/913424

**Published:** 2015-10-12

**Authors:** Anthony C. Ekennia, Damian C. Onwudiwe, Cyril Ume, Eno E. Ebenso

**Affiliations:** ^1^Department of Chemistry, Federal University Ndufu-Alike Ikwo, PMB 1010, Abakaliki, Ebonyi, Nigeria; ^2^Material Science Innovation and Modelling (MaSIM) Research Focus Area, Faculty of Agriculture, Science and Technology, North-West University (Mafikeng Campus), Private Bag X2046, Mmabatho, South Africa; ^3^Department of Chemistry, School of Mathematical and Physical Sciences, Faculty of Agriculture, Science and Technology, North-West University (Mafikeng Campus), Private Bag X2046, Mmabatho 2735, South Africa; ^4^Department of Chemical Engineering, Federal University Ndufu-Alike Ikwo, PMB 1010, Abakaliki, Ebonyi, Nigeria

## Abstract

A series of mixed ligand dithiocarbamate complexes with a general formula [ML_2_(py)_2_], where M = Mn(II), Co(II), Ni(II), and Cu(II), py = pyridine, and L = *N*-methyl-*N*-phenyl dithiocarbamate have been prepared and characterised by elemental analysis, FTIR and Uv spectroscopy, magnetic moment, and thermogravimetric and conductance analysis. The infrared spectra showed that symmetrical bidentate coordination occurred with the dithiocarbamate moiety through the sulfur atoms, while neutral monodentate coordination occurred through the nitrogen atom for the pyridine molecule in the complexes. The electronic spectra, elemental analysis, and magnetic moment results proved that the complexes adopted octahedral geometry. The conductance measurement showed that the complexes are nonelectrolytes proving their nonionic nature. The compounds were screened for three human pathogenic fungi: *Aspergillus flavus, Aspergillus niger*, and *Candida albicans*. The cobalt complex showed the best antifungal activity among the test compounds. Liquid-liquid extractive abilities of the ligand towards copper and nickel ions in different solvent media were investigated. The ligand showed a strong binding affinity towards the metals ions with an extractive efficiency of about 99%.

## 1. Introduction

Adduct formation (interaction of metal complexes with various ligating Lewis bases) allows the increase in coordination number of metal ions in a complex, while maintaining the same oxidation state [1–3]. Often, the physical properties of the resulting adducts are significantly different from their parent metal complexes, and this influences their biological activities [4–6]. The ability or tendencies of metal complexes to form adducts differ and are closely related to the geometry of the coordinated ligand, the ability of the Lewis base to accept *π*-electrons, and also the ionic size of the central metal ion [5]. The drift of electron density towards the sulfur atoms of dithiocarbamate bases, due to the mesomeric effect of the –NR_2_ group, influences the adduct formation of dithiocarbamate complexes [1, 3, 7]. When compared to other dithiolates such as the xanthate, the –NR_2_ group provides larger electrons towards the sulfur atoms resulting in the donation of electrons from the sulfur atoms to the nonbonding molecular orbital of the metal [8]. This decreases its availability for axial interaction with Lewis bases [9]. Subsequently, the conventional route of additional reaction which leads to adduct formation is not always favored. The properties of the R-group in the –NR_2_ moiety affects the stability and other physicochemical properties of a metal complex. Depending on the inductive effect (positive or negative) of the group(s) on the nitrogen atom, the flow of electrons towards the ligating CS_2_ group could either be reduced or enhanced [10, 11].

A number of dithiocarbamate adducts have been reported in literature [4, 12–18] with various geometries such as square planar [19], octahedral [20, 21] and trigonal prismatic [22]. Interestingly, their pyridine [1, 12, 13], 2,2′-bipyridine [13, 15] triphenylphosphine [10], and 1,10-phenanthroline [1, 15] adducts have been reported to possess antifungal properties. Some show activities against broad spectrum and are more active compared to their parent metal complexes and uncomplexed ligand [10].

Another important area where the property of dithiocarbamates has been employed in the environment is in the separation of metal ions via solvent extraction, due to the strong chelating ability toward inorganic species. Solvent extraction, involving chelation, has been reported as one of the most widely used techniques in the preconcentration and separation of metal ions from aqueous samples for analytical purposes due to its ease, speed, and wide scope [23, 24]. The technique has become more useful in recent years due to the development of selective chelating agents for trace metal determination [25–27]. In solvent extraction, a proper choice of extracting agents can achieve group separation or selective separation of trace elements with high efficiencies [25]. For example, sodium diethyldithiocarbamate can extract over 40 metal species from aqueous solutions into organic solvents [23]. The ability of these compounds to form complexes is responsible for their extensive use as analytical reagents of environmental importance [26]. The high tendencies of forming chelates with metal ions at very low concentrations (*μ*g/mL) make them versatile in the removal, in preconcentration, and also as extractive agents in the determination of toxic heavy metal ions at trace and ultratrace levels [25]. The dialkyldithiocarbamates have been reported to have poor extractive ability in an acidic environment (low pH) and are highly unstable [24, 26]. The half-life of 0.3 seconds of diethyldithiocarbamate at pH 2 describes the extreme instability of dialkyldithiocarbamate, and this hinders measurement in acidic environments. Monoalkyldithiocarbamates are more stable than dialkyldithiocarbamates in acidic solutions [26]. Hence, there is need to develop dithiocarbamate bases that will function as good preconcentration and extractive agents in different environment (acidic and basic) media.

Our earlier work was concerned with the evaluation of the antibacterial properties of some transition metal complexes of* N*-methyl-*N*-phenyl dithiocarbamate, which were found to be promising as antibacterial agents [28]. Considering the wide spread interest in dithiocarbamate compounds [29–31] and the interesting properties which occurs due to the change in the coordination number by the addition of Lewis bases to already existing square planer/tetrahedral metal complexes, it was considered of interest to study the antimicrobial properties of some new pyridine adducts of transition metal dithiocarbamate complexes. However, our study showed that while these adducts (unlike their precursor complexes in the reported study [28]) displayed no antibacterial properties, their antifungal activities are very good. Herein, we report the synthesis, characterization, and antifungal activities of the pyridine adducts of* N*-methyl-*N*-phenyl dithiocarbamate complexes of Mn(II), Co(II), Ni(II), and Cu(II). Furthermore, we report, for the first time, the extractability of Ni(II) and Cu(II) ions by* N*-methyl-*N*-phenyl dithiocarbamate from aqueous phase into the organic phase. The nature of the extracted compound was also investigated using Job's method of continuous variation.

## 2. Experimental

### 2.1. Physical Measurement

All the chemical reagents used were of analytical grade and were used as received without further purification.* N*-methyl aniline, carbon disulfide, and sodium hydroxide were obtained from Sigma Aldrich. Pyridine, nickel(II) chloride hexahydrate, cobalt(II) chloride dihydrate, copper(II) nitrate trihydrate, anhydrous copper(II) sulphate, manganese(II) nitrate hexahydrate, nickel(II) nitrate hexahydrate, pH tablets 1–10, ammonia, distilled water, hydrochloric acid, ammonia, and chloroform were obtained from Merck Co. Infrared spectra were recorded on a Bruker alpha-P FT-IR spectrometer in the 500–4000 cm^−1^ range. Microanalyses were carried out with a Fisons elemental analyzer. The thermal behaviour of the compounds was studied using an SDTQ 600 thermogravimetry analyser. Magnetic susceptibilities measurements were carried out on a Johnson Matthey magnetic susceptibility balance, and diamagnetic corrections were calculated using Pascal's constant. Conductivity measurements were conducted using a MC-1, Mark V conductivity meter with a cell constant of 1.0. UV-Vis spectra obtained on a Perkin Elmer Lambda 40 UV-Vis spectrometer as solid reflectance. Atomic absorption spectrometer (Hitachi-18050) was used in present work.

### 2.2. Synthesis of Compounds

The reaction was carried out in two stages which involved the reaction of the pyridine with the hydrated metal salts, followed by a displacement reaction with the dithiocarbamate ligand.

#### 2.2.1. Synthesis of Sodium* N*-Methyl-*N*-phenyl Dithiocarbamate [NaL]

Sodium* N*-methyl-*N*-phenyl dithiocarbamate was prepared according to the published procedure [28]. A solution of sodium hydroxide (8 g, 0.2 mol) in 10 mL of distilled water was prepared in a two-necked flask with a thermometer. To this solution, 21.80 mL of N-methyl aniline (density 0.985) was added and the mixture was stirred for approximately 2 h at a low temperature range of 2–4°C. The yellowish-white solid product which separated out was filtered, washed with small portions of ether, and recrystallized in acetone.

#### 2.2.2. Synthesis of MCl_2_·py_2_ Complexes

The synthesis was carried out according to the reported procedure [29]. 15 mL methanol solution of pyridine was added to a 25 mL solution of the respective hydrated metal(II) chlorides in methanol in 1 : 2 molar ratios. The mixture was refluxed for 1 h, and the solution was left to evaporate off the solvent. The precipitates obtained were collected, washed several times with methanol-water mixture to remove unreacted starting materials, and dried* in vacuo* [30].

#### 2.2.3. Synthesis of the [ML_2_(py)_2_]

To a 20 mL solution of MCl_2_·py_2_ [where M = Mn(II), Co(II), Ni(II), and Cu(II)] in methanol (1 mmol) was added a 20 mL methanolic solution of sodium* N*-methyl-*N*-phenyl dithiocarbamate (NaL) (2 mmol) [31] with continuous stirring for about 60 min at room temperature. The precipitate formed was filtered, washed with methanol and water, and dried* in vacuo*. The synthesis procedure is presented in Scheme 2.

*[MnL*_*2*_ *(py)*_*2*_ *] yield (57%).* Selected IR, *υ* (cm^−1^): 1432 (C=N), 1263 (C_2_–N), and 920 (C=S). Electronic spectra (*λ*max in nm): 340 nm, 556 nm, and 650 nm. Anal. Calc. for C_26_H_26_N_4_S_4_Mn (577.71): C, 54.05; H, 4.54; N, 9.70; S, 22.20. Found: C, 54.28; H, 4.80; N, 10.12; and S, 22.42%.

*[CoL*_*2*_ *(py)*_*2*_ *] yield (64%)*. Selected IR, *υ* (cm^−1^): 1438 (C=N), 1262 (C_2_–N), and 912 (C=S). Electronic spectra (*λ*max in nm): 352 nm, 366 nm, 670 nm, and 762 nm. Anal. Calc. for C_26_H_26_N_4_S_4_Co (581.70): C, 53.68; H, 4.50; N, 9.63; S, 22.05. Found: C, 53.72; H, 4.85; N, 9.54; and S, 21.80%.

*[NiL*_*2*_ *(py)*_*2*_ *] yield (52%)*. Selected IR, *υ* (cm^−1^): 1440 (C=N), 1260 (C_2_–N), and 908 (C=S). Electronic spectra (*λ*max in nm): 341 nm, 650 nm, and 686 nm. Anal. Calc. for C_26_H_26_N_4_S_4_Ni (581.46): C, 53.71; H, 4.51; N, 9.64; S, 22.06. Found: C, 54.02; H, 3.98; N, 9.32; and S, 21.91%.

*[CuL*_*2*_ *(py)*_*2*_ *] yield (70%)*. Selected IR, *υ* (cm^−1^): 1444 (C=N), 1260 (C_2_–N), and 921 (C=S). Electronic spectra (*λ*max in nm): 355 nm, 539 nm, and 545 nm. Anal. Calc. for C_26_H_26_N_4_S_4_Cu (586.32): C, 53.26; H, 4.47; N, 9.56; S, 21.88. Found: C, 52.87; H, 4.42; N, 10.08; and S, 22.14%.

### 2.3. Extraction Procedure

Aqueous solutions containing 1.5 × 10^−3^ M metal salt in appropriate buffer of pH 1–10 were equilibrated with equal volumes of the chloroform and methanol solutions of the ligand 4 × 10^−4^ M by shaking in a mechanical shaker at 25°C. The methanol was used to dissolve the ligand due to its insolubility in chloroform. The aqueous and organic phases were well agitated, increasing the contact and homogenizing the concentrations of the different species in order that the complexation reaction would not be limited by diffusion. After agitation, the solutions were allowed to stand for 15 min. The solution was transferred to a separating funnel where the aqueous layer was allowed to separate from the organic layer and transferred into a volumetric flask. The extraction was repeated with chloroform (5 mL). The concentration of copper(II) and nickel(II) ions in the aqueous phase was determined by AAS and that of the organic phase from the difference was determined by considering the mass balance [25, 26].

#### 2.3.1. Procedure for Continuous Variation Method

0, 1, 2, 3, 4, 5, and 6 mL 4 × 10^−4^ M of nickel and copper salt solutions were pipetted, respectively, and transferred into different 50 mL volumetric flasks (7 numbers). An aliquot of 6, 5, 4, 3, 2, 1, and 0 mL 4 × 10^−4^ M of the ligand was added, respectively, in such a way that the mole fraction of solution remained constant. The solution was buffered to pH 10. Wavelength of maximum absorbance was noted against a blank, which appeared at 622 nm for copper solution and 640 nm for nickel solution. All the measurements were made at 25°C.

## 3. Results and Discussion

### 3.1. Synthesis

The reaction of pyridine with the respective metal salts in methanol solution afforded the pyridine complexes, which were used as the precursor compounds for the formation of the pyridyl adducts of the dithiocarbamate. Attempts to prepare pyridine adducts via the conventional method, which usually involves the introduction of Lewis base into the preformed dithiocarbamate complex, were not successful. All the compounds were stable at room temperature.

### 3.2. FTIR Spectroscopy

The IR spectra of all the compounds show (=C–H) stretching vibration, due to the aromatic ring around 3040 cm^−1^ [30], while the (–C–H) stretching vibration due to the CH_3_ group appeared around 2864 cm^−1^. A strong band exhibited between 1430 and 1444 cm^−1^ in the spectra of all the compounds is assigned to the thioureide bond. These peaks are shifted to lower regions compared to the compounds without the pyridine molecule, suggesting the addition of the Lewis base to the metal ion [2, 3, 6]. The reduction in stretching frequency of thioureide in the pyridine adducts arises because of the relatively decreased electron flow of the nitrogen lone pair of electrons towards the central metal ion. The additional pyridine molecules result in an increase in the electron density [2, 7, 8]. The* v*(CS_2_) vibration appeared between 910 and 920 cm^−1^, as a single band indicating a symmetrical bonding of the two sulfur atoms to the metal ion [32]. The bands due to the pyridine molecules appeared in the range of 1601–1608 cm^−1^ in all the compounds. Other peaks were masked by the dithiocarbamate ligands.

### 3.3. Thermal Studies

**Figure 1:**
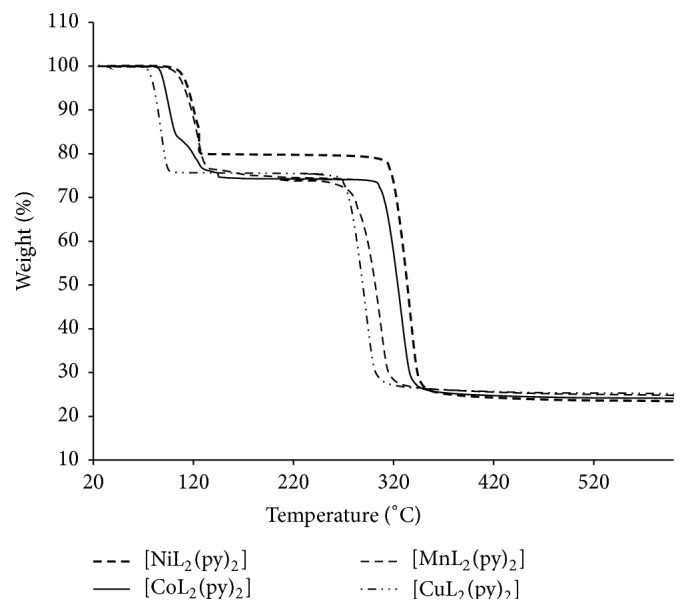
TGA graphs of the compounds under nitrogen flow.

The thermal property of the compounds was studied by TGA in the temperature range of 25 to 600°C, under nitrogen atmosphere, and presented in Figure 1. Thermogravimetry data reflect the thermal stability of compounds. The compounds showed very similar thermal behaviour. The decomposition occurred in two steps. The first degradation step is in the range of 65–95°C, and approximately 27% of weight loss was observed which corresponds to two pyridine groups in agreement with Siddiqi et al. report [33]. The second stage of decomposition is sharp, in the range of 275 to 350°C and involved the decomposition of the remaining organic moiety which constitutes approximately 43% of the remaining mass of the complex. The residue left behind corresponds to the respective metal sulfide. Finally, the thermogram maintained a straight line which did not change even though heating proceeded up to 600°C indicating that there is no further decomposition. Hence, respective metal sulfide is the end product of all the complexes. The thermal decomposition behaviour of the complexes appeared to be related to the size of the central metal ion. After the release of the pyridine group, the stability of the dithiocarbamate followed the order: Ni > Mn > Co > Cu. This observation is in agreement with the earlier report [28].

### 3.4. Electronic Spectra and Magnetic Moment

Electronic spectra of all the complexes were recorded as solid reflectance according to literature procedure [28]. Absorption bands are the result of electronic transitions within the ligands (*n* → *π* ^*∗*^, *π* → *π* ^*∗*^), metal d-d transitions, and charge transfer transitions (metal to ligands and ligand to metal charge transitions) [34, 35]. The bands around 340–366 nm in the metal adducts were due to the *n* → *π* transition from the C=N group of the pyridine molecule and dithiocarbamate moiety.

The electronic spectrum of the cobalt adducts showed absorption bands around 366 nm, 352 nm, 670 nm, and 762 nm. The bands around 352 and 366 nm are due to *n* → *π* ^*∗*^ transitions of the C=N group in the pyridine and the dithiocarbamate moiety [28, 36]. The d-d bands at 670 nm and 762 nm are due to ^4^T_1_g → ^4^A_2_g and ^4^T_1_g → ^4^T_1_g(p) transitions, respectively, for an octahedral d^7^ geometry with ^4^F ground term [37, 38]. The assignment of a low spin octahedral geometry to the cobalt adduct was corroborated with a magnetic moment value of 1.82 BM. In high spin octahedral cobalt complexes, the magnetic moment lies between 4.7 and 5.2 BM, while low spin octahedral cobalt complexes show magnetic moment from 1.70 to 1.85 BM [34, 39].

The nickel adduct showed bands at 341 nm, 650 nm, and 686 nm due to *n* → *π* ^*∗*^ transition in the UV region and visible bands for ^3^A_2_g → ^3^T_1_g(F) and ^3^A_2_g → ^3^T_1_g(P) transitions consistent with an octahedral d^8^ system in ^3^F ground term [34]. The ^3^A_2_g → ^3^T_2_g transition often tails into the infrared region and, thus, it is not observed in the visible region [40, 41]. The magnetic moment value of 2.78 BM corroborates an octahedral geometry; a magnetic moment of 2.90–3.30 BM is expected for an octahedral d^8^ system. The lower value may be due to antiferromagnetism. A tetrahedral d^8^ system has a magnetic moment of 3.20–4.10 BM, while a square planar nickel(II) complex will have a zero moment [40].

Octahedral d^9^ copper complexes are expected to have a broad spectrum due to ^2^Eg → ^2^T_1_g with a ^2^D ground term. However, due to Jahn-Teller effect caused by asymmetrical filling of the eg set of orbitals, a splitting of ^2^Eg and ^2^T_2_g into ^2^B_1_g, ^2^A_2_g, and ^2^B_2_g, ^2^Eg, respectively, is usually observed [42]. Hence, the transitions may lie within one envelope or may resolve into two or three absorption band components. The ligand bands for the copper adduct were observed at 355 nm and assigned to *n* → *π* ^*∗*^ transition. The d-d transition was observed at 539 nm and 545 nm due to Jahn-Teller effect. The magnetic moment for a mononuclear copper complex irrespective of geometry is 1.9–2.2 BM [38]. The copper adduct had a magnetic moment of 2.02 BM which collaborates its mononuclear nature.

The Mn(II) adduct showed three weak bands at 340 nm, 556 nm, and 650 nm; one ligand band and two forbidden transition bands typical of 6-coordinate octahedral geometry, and was assigned to *n* → *π* and the forbidden transitions of ^6^A_1_g → ^4^T_1_g and ^6^A_1_g → ^4^T_2_g (G). The effective magnetic moments of Mn(II) complexes are expected to be close to the spin-only value of 5.90 BM Since the ground term is ^6^A_1_g, there is no orbital contribution. Consequently, a moment of 5.71 B.M. observed for this complex indicates that it is high spin and complementary of octahedral geometry [36, 43].

### 3.5. Molar Conductivity Measurement

The results of the elemental analysis are in good agreement with the calculated values. The metal contents of the complexes were determined according to literature methods [28]. The electrolytic nature of the complexes was measured in DMSO at 10^−3^ M concentration. The molar conductivity values were 19.16 Ω^−1^cm^2^ mol^−1^, 13.06 Ω^−1^cm^2^ mol^−1^, 24.4 Ω^−1^cm^2^ mol^−1^, and 10.84 Ω^−1^cm^2^ mol^−1^ for NiL_2_Py_2_, CuL_2_Py_2_, CoL_2_Py_2_, and MnL_2_Py_2_. The results show that the complexes were nonelectrolyte in nature [44, 45].

### 3.6. Antifungal Screening

**Figure 2:**
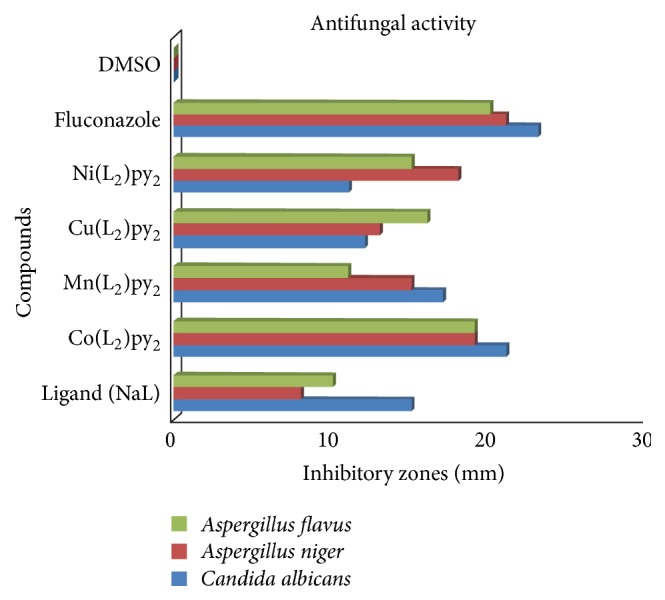
A histogram representative of antifungal activities of the pyridine adducts.

*Disc Diffusion Method*. A disc application technique was employed* in vitro* to evaluate the antifungal activities of the pyridine adducts [46]. The fungi used for the screening were* Candida albicans,* a diploid fungus and a causal agent of opportunistic oral and genital infections in humans;* Aspergillus flavus*, a mold type fungal strain which may invade arteries of the lung or brain to cause infections and also produce a toxin; and* Aspergillus niger* which is one of the most common causes for otomycosis [10]. Mature conidia of fungal isolates were harvested from potato dextrose agar (PDA) plates and suspended in ringer solution. Conidial suspension (1 mL) representing each fungal isolate was then spread on a 90 mm Petri dish containing PDA (20 mL) with the excess of the conidial suspension decanted and allowed to dry. The compounds 100 *μ*g/Ml were dissolved in dimethyl sulfoxide (DMSO). Sterile 6 mm diameter test discs were impregnated with 15 *μ*L of the solution of each test compound to contain 100 *μ*g/disc in triplicate. Fluconazole (100 *μ*g/disc) was used as a reference drug for fungal inhibition, while DMSO was used as a negative control. Plates were incubated at room temperature for 24 h. The radius of the inhibition zone of fungal growth was measured after 1 day and expressed as the inhibition zone in mm. The results are presented in Table 1, and Figure 2 is the histogram representation of the activities.

The screened compounds displayed good* in vitro* antifungal inhibitory properties at the experimental concentration of 100 *μ*g/mL. The complexes showed improved antifungal inhibition compared to the uncomplexed ligand. This may be attributed to the increased toxicity of the complexes due to the presence of the pyridine molecules and the metal ions. The cobalt adduct showed the best inhibitory properties among the test compounds and was 91% as active as fluconazole against* Candida albicans*. The screened compounds were all active against* Aspergillus *spp. The cobalt complex was 90% and 95% as active as fluconazole against* A. niger* and* A. flavus,* respectively, thereby proving its usefulness as lead compounds in broad spectrum research for fungal organisms. Antibacterial study of the compounds involving Gram positive and Gram negative bacteria was carried out* in vitro*. The complexes showed no toxicity towards the various bacteria used. The absence of antibacterial activities observed in these compounds could be ascribed to the poor permeability of the compounds through the bacterial cell wall, or lack of available coordination sites due to the octahedral geometry of the adducts.

Dithiocarbamates have been reported to react with HS containing enzymes and coenzymes of fungal cells to form isothiocyanates, which is fungitoxic in nature [47–49]. Enzyme inhibition could also be possible by complex formation of dithiocarbamate ligand with metal atoms of metal containing enzymes [50]. Some dithiocarbamate compounds like* N*,*N*-dimethyldithiocarbamates penetrate the cell membrane of the fungi before it is reduced to isothiiocyanates to exact its antifungal effect [51]. However, the mechanism of the action of metal complexes of dithiocarbamate is not fully understood. According to some authors, metal complexes react with copper containing proteins or enzymes and block them from being functional. This reaction depends on the degree of stability of the newly formed compound compared to the former. The copper ion in the protein or enzyme will replace the metal ion of the complex forming a biomolecular complex, thereby disrupting the normal functioning of the protein or enzyme in the fungi. The more stable the enzyme-complex, the higher its fungicidal effect [52].

Metal complexes of 2 : 1 metal to ligand ratio are often considered nontoxic to fungi due to their very low water solubility which makes it difficult to penetrate the cell membrane. Inconsistency in the assertion according to some authors is due to the conversion of such complexes to a 1 : 1 ratio within the fungi cells [52]. That might possibly be the reason why the metal complexes of* N*-methyl-*N*-phenyl dithiocarbamate reported in our previous work [28] showed no antifungal activity. However, the mode of action of adducts of dithiocarbamate complexes is an unsettled question. In order to understand the functions responsible for antifungal activities of pyridine adducts of dithiocarbamate complexes, more studies are needed to be carried out with a series of analogous ligands and their complexes against a series of phytopathogenic fungi.

### 3.7. Solvent Extraction Studies

On addition of the ligand to the aqueous phase, it reacts with copper and nickel to form colored metal complexes which transfer from the aqueous phase to the organic phase. Maximum color development occurred within 3 min (Scheme 2). The copper solution turned reddish-brown and the nickel solution turned greenish on the addition of the ligand. The ligand's metal ion extraction was studied with the simultaneous determination of Ni^2+^ and Cu^2+^ in aqueous phase at different pH levels. The obtained results are expressed in terms of the extraction ratio *R*(%) and the partition coefficient *D* (Tables 2 and 3) as follows:

(1) R%=Mi−MfMi×100,D=Mi−MfMi,

where [*M*_*i*_] and [*M*_*f*_] are the initial and final concentrations in the aqueous phase.

The degree of separation was determined in terms of separation factor, *S*_*f*_, defined as the ratio of distribution ratio for nickel ion to the distribution ratio for copper ion:

(2) $$\begin{array}{c} S_{f}=\frac{D_{Ni}}{D_{Cu}}. \end{array}$$

#### 3.7.1. Effect of pH on Extraction

**Figure 3:**
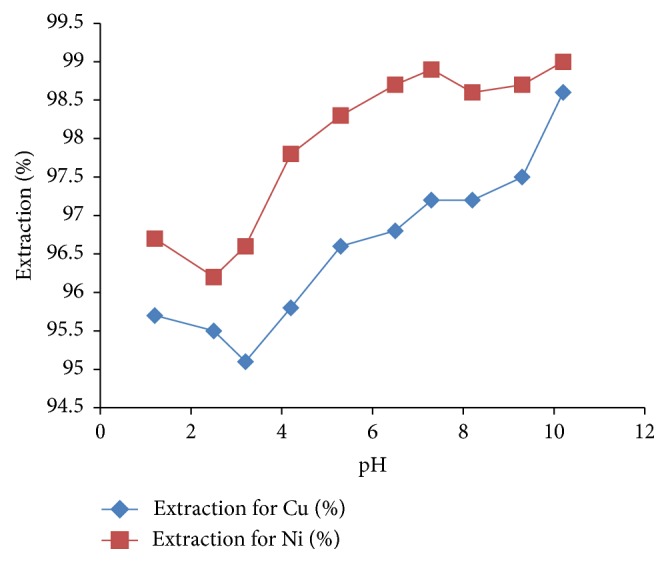
Graph of effect of pH on the extraction of copper ion or nickel ion extraction by* N*-methyl-*N*-phenyl dithiocarbamate.

The extractability and selectivity of metal ions were evaluated as a function of pH. The pH of the aqueous solution before extraction was varied from 1.2 to 10.2 by using solutions of HNO_3_ (0.1 M) and NaOH (0.1 M). The percentage extraction and distribution ratio are described in Tables 2 and 3, and the graphical presentation is given in Figure 3.

The highest extractability and selectivity for Ni^+2^ and Cu^+2^ were achieved at pH 10. The separation factor of Ni^2+^ to Cu^2+^ at aqueous solution of pH 10 is 1, making* N*-methyl-*N*-phenyl dithiocarbamate anion a poor reagent in the selective separation of nickel and copper ions in solution.

The solvent extraction process may be represented by the following:

(3) $$\begin{array}{c} {M^{2+}}_{\left(aq\right)}+nNaL_{\left(org\right)}⟷MLn_{\left(org\right)}+{{nNa}^{+}}_{\left(aq\right)} \end{array}$$

where NaL = the extractive reagent, *n* = number of moles, M = Cu, and Ni is ions. The subscript (aq) and (org) denote the aqueous and organic phases, respectively.

**Scheme 1:**
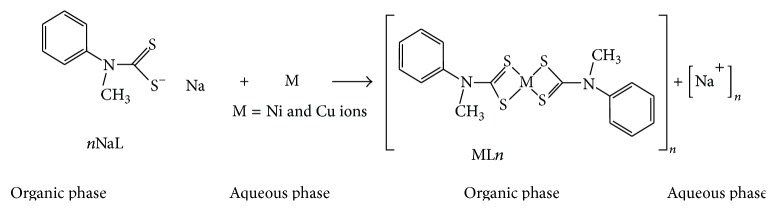
Schematic representation of the extraction process.

**Scheme 2:**
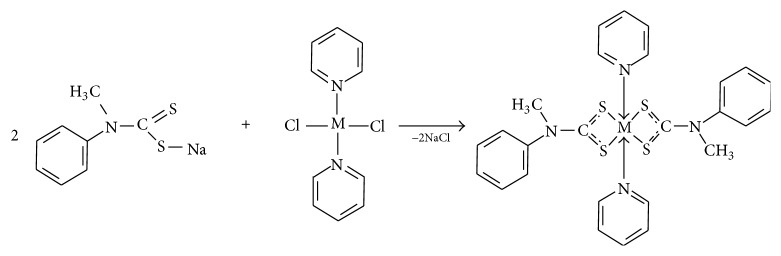
Synthesis procedure.

The extraction mechanism corresponds to a cation exchange, in which a complex of stoichiometric formula (CuL_*n*_ and NiL_*n*_) is formed in the organic phase liberating, at the same time, *n* moles of Na^+1^ ions in the aqueous phase [26] as presented in Scheme 1.

The results presented in Tables 2 and 3 showed that the ligand had the highest percentage extraction at a pH of 10, with 99.0% Ni and 98.6% Cu ions extracted from the aqueous phase into the organic phase. Interestingly, the ligand also recorded a very good percentage extraction values at an acidic pH (1–5). This makes the ligand suitable for solvent extraction study in both acidic and basic media.

#### 3.7.2. Nature of the Specie in the Organic Phase at pH 10

**Figure 4:**
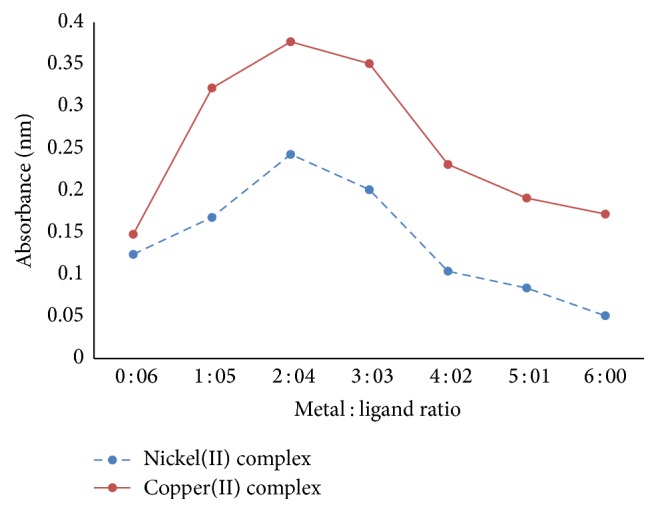
Job's curves of equimolar solutions at 622 nm copper ion and 640 nm nickel ion extraction.

The nature of the extracted species in the organic phase at the pH with the best extractive potentials by the ligand (pH 10) was investigated using Job's method of continuous variation. The results are presented in Table 4 and Figure 4. According to the results, the metal to ligand ratio of 2 : 4 gave the maximum absorbance for both nickel and copper. This implies that the species in organic phases at pH 10 are four coordinate complexes of bis(*N*-methyl-*N*-phenyl dithiocarbamate) copper(II) and bis(*N*-methyl-*N*-phenyl dithiocarbamate) nickel(II), respectively, which is in agreement with the reported stoichiometry [28].

## 4. Conclusions

In conclusion, a series of pyridine adducts of* N*-methyl-*N*-phenyl dithiocarbamate were synthesized, and their structure has been established on the basis of spectral studies, magnetic moment measurements, and elemental analysis. The compounds and the dithiocarbamate ligand were evaluated for antifungal properties against three important pathogens, namely,* Aspergillus flavus, Aspergillus niger,* and* Candida albicans*. The adducts displayed better antifungal inhibitory activity than the dithiocarbamate ligand. Cobalt complex showed the highest activity among the test compounds. Metal ions extraction studies showed that the ligand has strong extractive ability towards Cu and Ni ions in both acidic and basic medium, with its best condition at pH 10. The selective separation of the ligand for Cu and Ni from aqueous solution was small, indicating that the ligand is not an ideal compound for separating these two metals in a mixture of both. The adducts have potentials as lead compounds in broad spectrum research for antifungal agents, and the ligand is a good reagent for preconcentration and extraction of metal ions in different media.

## Figures and Tables

**Table 1:** Antifungal activities of the pyridine adducts of *N*-methyl-*N*-phenyl dithiocarbamate.

Compounds	*Candida albicans*	*Aspergillus niger*	*Aspergillus flavus*
Ligand (NaL)	15 ± 0.7	8 ± 1.4	10 ± 0.7
Co(L_2_)py_2_	21 ± 0	19 ± 0.4	19 ± 0.2
Mn(L_2_)py_2_	17 ± 0.2	15 ± 0.7	11 ± 0.4
Cu(L_2_)py_2_	12 ± 0.7	13 ± 0	16 ± 0.2
Ni(L_2_)py_2_	11 ± 0	18 ± 0.2	15 ± 1.5
Fluconazole	23 ± 0	21 ± 0.2	20 ± 0
DMSO	—	—	—

**Table 2:** Extraction efficiency of nickel ion at different pH levels.

pH	Amount of metal found in the aqueous phase *μ*g/mL	Amount of metal in the organic phase *μ*g/mL	Distribution ratio	Percentage extraction %
1.2	2.95	85.86	0.967	96.7
2.5	3.42	85.39	0.962	96.2
3.2	3.04	85.77	0.966	96.6
4.2	1.96	86.85	0.978	97.8
5.3	1.52	87.29	0.983	98.3
6.5	1.14	87.67	0.987	98.7
7.3	1.02	87.79	0.989	98.9
8.2	1.21	87.60	0.986	98.6
9.3	1.18	87.63	0.987	98.7
10.2	0.86	87.95	0.990	99.0

**Table 3:** Extraction efficiency of copper ion at different pH levels.

pH	Amount of metal found in the aqueous phase *μ*g/mL	Amount of metal found in the organic phase *μ*g/mL	Distribution ratio	Percentage extraction %
1.2	4.08	91.40	0.957	95.70
2.5	4.29	91.19	0.955	95.50
3.2	4.63	90.85	0.951	95.10
4.2	3.99	91.49	0.958	95.80
5.3	3.27	92.21	0.966	96.60
6.5	3.05	92.43	0.968	96.80
7.3	2.68	92.80	0.972	97.20
8.2	2.71	92.77	0.972	97.20
9.3	2.42	93.06	0.975	97.50
10.2	1.32	94.16	0.986	98.60

**Table 4:** Experimental data for nickel(II) and copper(II) extraction by Job's continuous variation method.

Number	Metal : ligand mL	Absorbance for nickel(II) complex	Absorbance for nickel(II) complex
1	0 : 6	0.124	0.148
2	1 : 5	0.168	0.322
3	2 : 4	0.243	0.377
4	3 : 3	0.201	0.351
5	4 : 2	0.104	0.231
6	5 : 1	0.084	0.191
7	6 : 0	0.051	0.172
